# Reducing the Effect of Spurious Phase Variations in Neural Oscillatory Signals

**DOI:** 10.3389/fncom.2018.00082

**Published:** 2018-10-08

**Authors:** Zeinab Mortezapouraghdam, Farah I. Corona-Strauss, Kazutaka Takahashi, Daniel J. Strauss

**Affiliations:** ^1^Systems Neuroscience & Neurotechnology Unit, Faculty of Medicine, Saarland University, Homburg, Germany; ^2^School of Engineering, Saarland University of Applied Sciences, Saarbruecken, Germany; ^3^Research Computing Center and Organismal Biology and Anatomy, University of Chicago, Chicago, IL, United States; ^4^Leibniz-Institute for New Materials, Saarbruecken, Germany

**Keywords:** instantaneous phase, spurious phase, Kalman smoother, phase synchronization, phase reset

## Abstract

The phase-reset model of oscillatory EEG activity has received a lot of attention in the last decades for decoding different cognitive processes. Based on this model, the ERPs are assumed to be generated as a result of phase reorganization in ongoing EEG. Alignment of the phase of neuronal activities can be observed within or between different assemblies of neurons across the brain. Phase synchronization has been used to explore and understand perception, attentional binding and considering it in the domain of neuronal correlates of consciousness. The importance of the topic and its vast exploration in different domains of the neuroscience presses the need for appropriate tools and methods for measuring the level of phase synchronization of neuronal activities. Measuring the level of instantaneous phase (IP) synchronization has been used extensively in numerous studies of ERPs as well as oscillatory activity for a better understanding of the underlying cognitive binding with regard to different set of stimulations such as auditory and visual. However, the reliability of results can be challenged as a result of noise artifact in IP. Phase distortion due to environmental noise artifacts as well as different pre-processing steps on signals can lead to generation of artificial phase jumps. One of such effects presented recently is the effect of low envelope on the IP of signal. It has been shown that as the instantaneous envelope of the analytic signal approaches zero, the variations in the phase increase, effectively leading to abrupt transitions in the phase. These abrupt transitions can distort the phase synchronization results as they are not related to any neurophysiological effect. These transitions are called spurious phase variation. In this study, we present a model to remove generated artificial phase variations due to the effect of low envelope. The proposed method is based on a simplified form of a Kalman smoother, that is able to model the IP behavior in narrow-bandpassed oscillatory signals. In this work we first explain the details of the proposed Kalman smoother for modeling the dynamics of the phase variations in narrow-bandpassed signals and then evaluate it on a set of synthetic signals. Finally, we apply the model on ongoing-EEG signals to assess the removal of spurious phase variations.

## 1. Introduction

The assessment of voltage changes of measured neural activities in terms of their level of *synchronization* has been one of the main evaluation methods for understanding the behavior of numerous cognitive processes and biological systems (Tass et al., 1998; Lachaux et al., 1999; Rosenblum et al., 2001; Fell and Axmacher, 2011; Park and Rubchinsky, 2012; Mortezapouraghdam et al., 2014, 2016; Thounaojam et al., 2014; Watanabe et al., 2015; Noda et al., 2017). Phase synchronization is observed from a single cell recording where groups of neurons from the same or different populations fire simultaneously, up to a larger scale where different regions of the brain exhibit a synchronized and coherent behavior in their neural activities (Siapas et al., 2005; Laine et al., 2012). Analyzing the phase of neural activities from invasive to non-invasive scales has been found to be informative for decoding the underlying neural mechanisms, understanding the propagation of neural firing and thereby giving us insights on the association between different neural assemblies (Lutz et al., 2002; Nolte et al., 2004; Busch et al., 2009; Uhlhaas et al., 2009; Canavier, 2015; Voloh and Womelsdorf, 2016).

The coherent activity in neural population can be observed among different neural assemblies for different cognitive and motor tasks. It has been particularly used to study the effect of cognitive binding with regards to different stimulations (Makeig et al., 2002; Strauss et al., 2005; Klimesch et al., 2007). For example, in Mortezapouraghdam et al. (2015) the level of *phase alignment* has been used to differentiate between different processes of habituation and non-habituation. In Bernarding et al. (2013) phase synchronization has been used to objectively determine the level of selective attention to auditory stimulations. Abnormal activities in phase synchronization of neural oscillators have been investigated across different spectrums of neuropsychiatric disorders such as schizophrenia and attention deficit disorders (Tcheslavski and Beex, 2006; Bob et al., 2008). Epilepsy is associated with a hypersynchronous state of neuronal activities across the brain. Using the phase synchrony techniques, different approaches for prediction of epileptic episodes have been proposed (Zheng and Voight, 2006).

**Figure 1:**
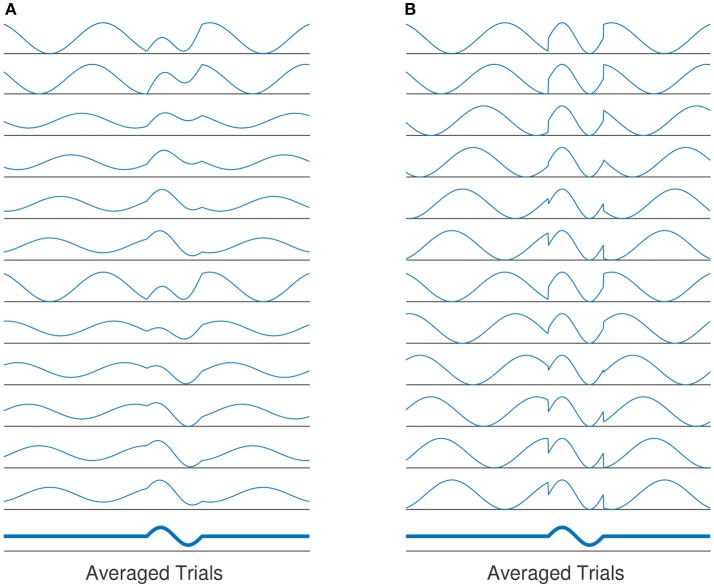
**(A)** The evoked classical model assumes that the evoked components of averaged ERP is generated by a constant evoked response that is added onto EEG activity. The evoked responses have the same latency and polarity for all trials. The average of all responses over all trials will yield the averaged ERP. **(B)** Another explanation of ERP genesis model, the average evoked response is based on phase-resetting of background EEG activity. Averaging over all trials by which phase reset has occurred, the same results as in **(A)** is produced.

The role of phase synchronization of ongoing oscillatory activities has been deeply discussed in the area of ERP generation as well (Min et al., 2007). The *phase modulation* view of ERP genesis states that the generation of evoked-related potential (ERP) is not independent from the background (ongoing) EEG activities (see Sayers et al., 1974; Yeung et al., 2004 and the reference therein for more details). It is assumed that ERPs are generated by the re-organization of stimulus induced phase resets of ongoing EEG rhythms (Sayers et al., 1974; Makeig et al., 2002; Penny et al., 2002). Thereby, the ERP generation is not solely based on superposition of evoked, fixed-latency and fixed-polarity responses that are independent from the ongoing EEG activity (Kolev and Yordanova, 1997; Sauseng et al., 2007). Based on this definition, the background EEG activity comprise an important part of the ERP generation process. This view is also referred to as *phase modulation* (PM), in contrast to the classical view, namely the *amplitude modulation*(AM). Figure 1 illustrates the classical view of ERP generation against the phase-resetting model.

Given the importance and the broad applicability of phase synchronization across different domains, applying proper tools and techniques for measuring the level of phase synchrony of neural activities is crucial. Phase locking value (PLV) is among the most common approaches used for measuring the phase synchrony (Rosenblum et al., 2000; Hurtado et al., 2004; Park and Rubchinsky, 2012; Aydore et al., 2013). Empirical mode decomposition (EMD), frequency flow analysis (FFA), phase-amplitude coupling and event-related synchronization are among examples of methods used for quantifying the level of phase synchronization among different neural oscillatory activities (Lachaux et al., 1999; Rudrauf et al., 2006; Dvorak and Fenton, 2014). In Mortezapouraghdam et al. (2014, 2016), a Bayesian framework has been used to detect the significant changes in the phase synchronization level. Thereby, a *reliable phase estimation* is important for obtaining a realistic and stable estimate of the level of phase synchrony and coherency of neural activities.

Removal of *artificial phase variations* in the signal is one of the important steps for obtaining a reliable measure of phase synchronization. Spurious phase variation in this context refers to *phase resets* which are not related to any underlying neurophysiological activities. In the recent study by Sameni and Seraj (2016, 2017), one of the effects that lead to artificial phase variations in EEG signals has been thoroughly explained. It has been demonstrated that the *low envelope* of an analytical signal after narrow band-passing can cause *abrupt changes* in the instantaneous phase (IP) or instantaneous frequency (IF) of the signal. As a consequence, these spurious phase variations can be falsely correlated and interpreted as a response to stimulations or cognitive activities and distort the results. These artificial phase alignments are called *spurious phase-resets*. In Sameni and Seraj (2016), a robust measure based on a Monto-carlo estimation scheme has been proposed for computing a more reliable estimate of the phase.

As a contribution to the proposed method in Sameni and Seraj (2016) and Seraj and Sameni (2017) and the concept of removal of noisy phase resets, we present a model that is able to *remove spurious phase variations* in the IP component of signals by modeling the behavior of IP over time. Our proposed method is a special case of a Kalman smoother (KS), which is applied after applying a set of different narrow-bandpassed filters with slight parameter variations for a robust estimation [an initial study of the proposed methodology has been published in Mortezapouraghdam and Strauss (2017)]. The IP and IE are modeled using a multivariate complex Gaussian distribution and illustrate how the information in the IE of narrow-bandpassed signals can contribute to elimination of spurious phase jitters.

The organization of the paper is as follows: We first describe the proposed methodology in detail and test it on synthetic data with spurious phase variations. The method is evaluated using different signal-to-noise (SNR) ratios. We apply the proposed method on examples of ongoing EEG measurements to examine the applicability of the model to real measurements^1^. Finally, we discuss the setting of the KS parameters and its potential use for future studies.

## 2. Materials and methods

In this section, we briefly explain the effect of spurious phase variation followed by describing the proposed approach for removing the phase slips. In the second part of this section, the experimental setting and the measuring procedures are explained.

### 2.1. Phase singularities: definition of spurious phase slips and types

One of the main steps before extraction of IP is the filtering process. To obtain a meaningful interpretation of phase modulations, the signal is narrow-bandpassed (Chavez et al., 2006; Sameni and Seraj, 2017). A FIR (finite impulse response) filter is usually applied to avoid the distortion of phase information (see chapter 5 of Handy, 2005 for more references). The IP is obtained by computing the analytic signal, commonly by either applying a wavelet transformation (WT) or Hilbert transformation (HT). The HT of a signal *x*(*t*) is represented as

$$\begin{array}{l} \hat{x}\left(t\right)=x\left(t\right)+iH\left(x\left(t\right)\right)=x\left(t\right)+iu\left(t\right). \end{array}$$

such that a real-valued signal is converted to a complex plane using HT. Measures such as the IE and IP are computed from $A_{t}=\sqrt{x{\left(t\right)}^{2}+u{\left(t\right)}^{2}}$ and $\theta \left(t\right)=\tan^{-1}\left(\frac{u\left(t\right)}{x\left(t\right)}\right)$ respectively, where θ ∈ [−π, π), *t* ∈ ℝ. The IP transits to −π when it reaches π. One of the techniques for tracking the modulations of IP in long time intervals is analyzing the unwrapped version of the IP. The unwrapped IP is obtained by adding 2π every time a reset occurs. We use *p*:ℝ → ℝ^+^ for unwrapped phase. Using the unwrapped phase, the angular frequency is defined as $z\left(t\right)=\frac{p_{t}-p_{t-1}}{\Delta t}$ with Δ*t* being the sampling period.

**Figure 2:**
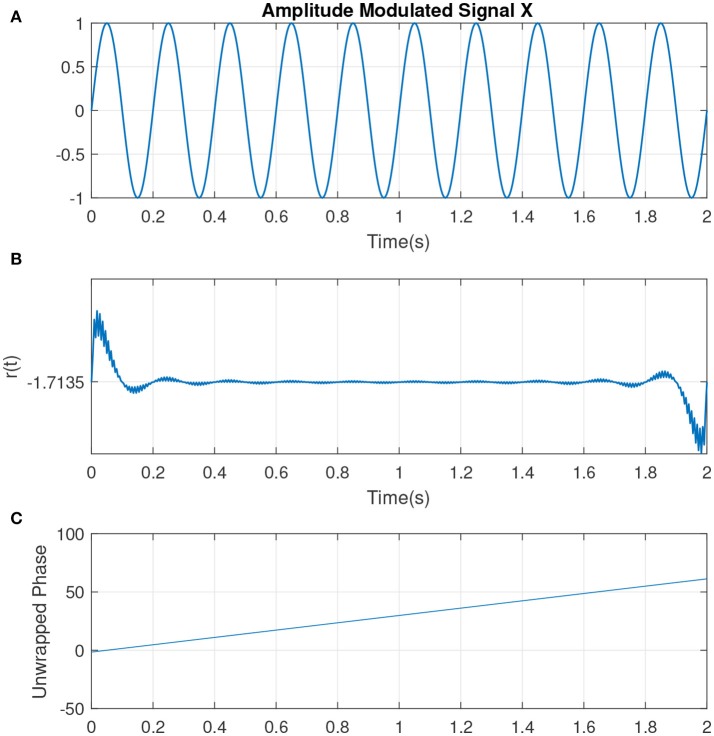
**(A)** An example of a sine wave *X* = sin(ω_0_*t*) with a center frequency of *f*_*c*_ = 5Hz or ω_0_ ≈ 31 (rad/s). **(B)** The residual *r*(*t*). As there are no phase shifts in the signal, no jumps is observed in *r*(*t*). The small jitters at the beginning correspond to the filtering. **(C)** The unwrapped phase that is reflecting the same interpretation as in **(B)**.

**Figure 3:**
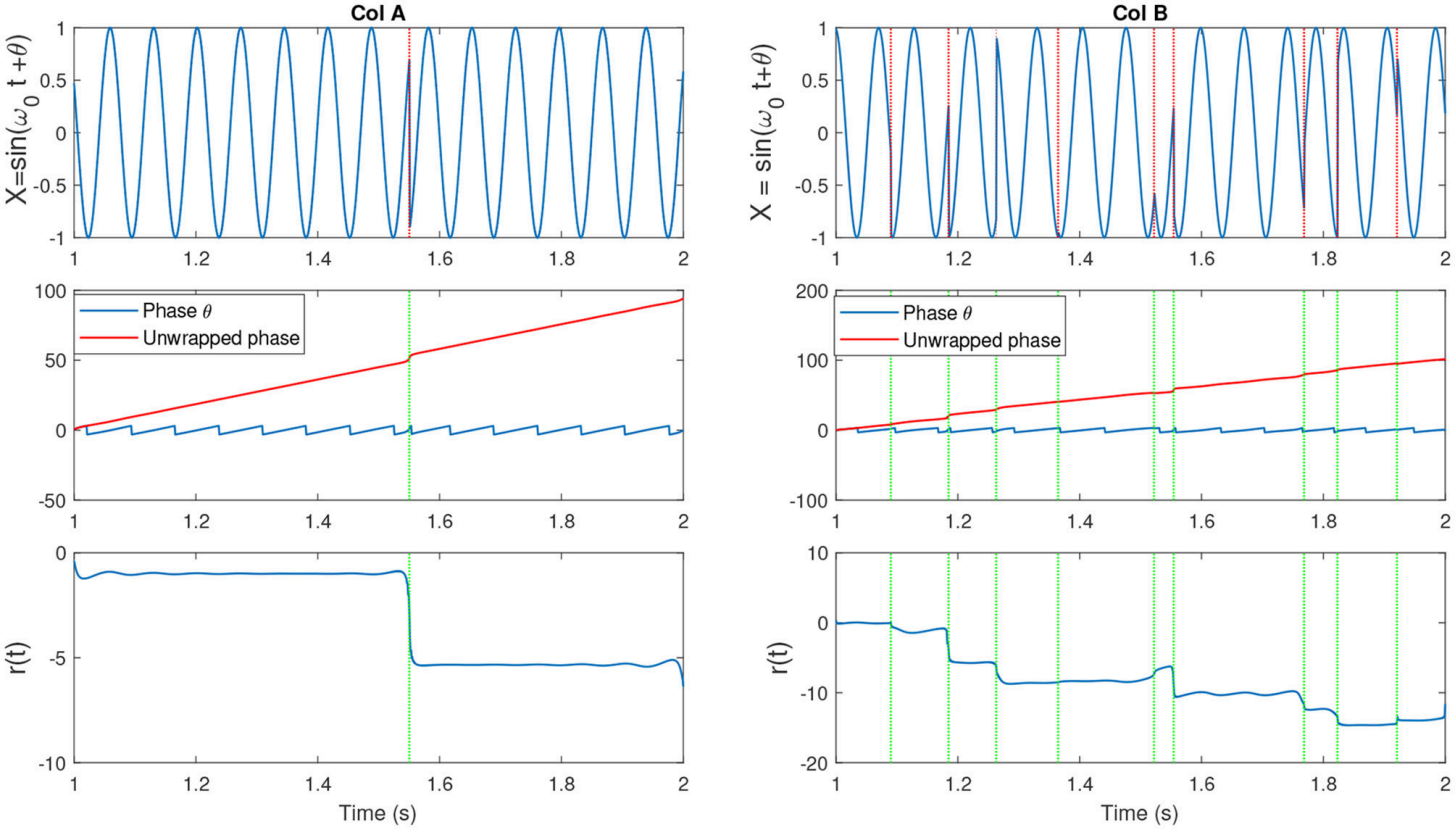
(Col A, **top**) An example of a sine wave *X* = sin(ω_0_*t* + θ) with *f*_*c*_ = 7. We introduced one artificial shift in the signal. **(Middle)** The unwrapped IP of X [denoted as (*p*(*t*)] and the wrapped version θ(*t*). **(Bottom)** The residual *r*(*t*) which is computed as *r*(*t*) = *p*(*t*) − ω_0_*t*. The shift in the signal has been shown as an abrupt jump in the residual. (Col B, **top**) An example of a sine wave with more random shifts in the signal. Corresponding phase and residual, similar to previous plot have been illustrated. Figure with some modifications has been adapted from Mortezapouraghdam and Strauss (2017).

The slope of *p*(*t*) is related to the *mean frequency*. The slope of a signal with center frequency *f*_*c*_ which best determines the activities at that particular frequency is given by ω_0_ = 2π*f*_*c*_ (measured in *rad*/*sec*). If the IP contains no additional resets, it is uniformly distributed. This can represented by a sine wave with complete cycle, at which the IP is uniformly distributed (see Figure 2, illustrating the simplest condition along with the parameters such as unwrapped phase and residual defined earlier as indicators for the variations in the phase of the signal). Hence the change to the uniformity can be represented by the difference between *p*(*t*) and the line ω_0_*t*, i.e., *r*(*t*) = *p*(*t*) − ω_0_*t*. See Figure 3 which is an example of two sine waves with high and low number of resets along with *r*(*t*).

#### 2.1.1. Spurious phase variation

After applying a narrow band-pass filter to the measured EEG oscillations, the resulting *x*_*t*_, θ_*t*_ can be described by the additive model of

$$x_{t}=X_{t}\cos\left(\omega_{0}t+\theta_{t}\right)+W_{t}=s_{t}+W_{t}$$

where θ_*t*_ is the residual phase *r*(*t*), $\omega_{0}=\frac{2\pi f_{c}}{f_{s}}$, and *X*_*t*_ is the envelope. It is assumed that the EEG signal is a superposition of background activities (considered as noise) and the desired neural activity. The noise or the background EEG is modeled by *W*_*t*_ and it is assumed to be Gaussian distributed $W_{t}\sim N\left(0,\sigma^{2}\right)$. The specific desired neural activity is denoted as *s*_*t*_. The IP θ_*t*_ (θ_*t*_ ∈ [−π, π)) and the envelope *X*_*t*_ of the narrow-bandpassed signal are slowly varying functions of *t*. Hence, it is expected that the changes between θ_*t*_ and θ_*t* + 1_ to be small.

**Figure 4:**
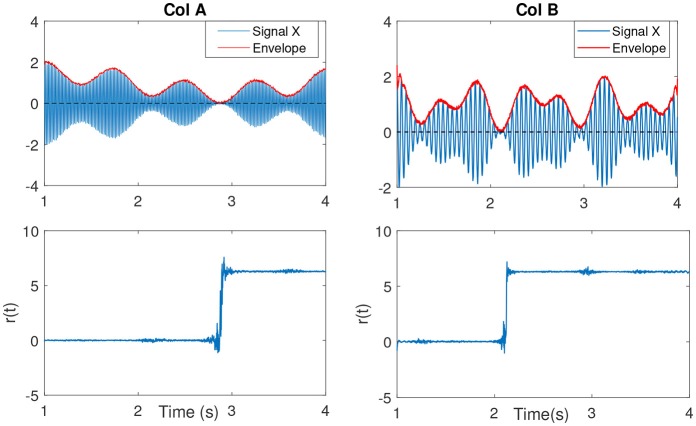
(Col A, first plot) An example of an amplitude modulated signal with a mean frequency *f*_*c*_ = 50*Hz*. Red line shows the IE. The second plot shows *r*(*t*). As the IE of the signal approaches to zero, the phase variations increase, best seen as an abrupt change. This is clearly evident in comparison to the other envelope values throughout the signal at other time samples. (Col B, first plot) The same explanation as in Col A, however with a lower mean frequency signal *f*_*c*_ = 15*Hz*.

**Figure 5:**
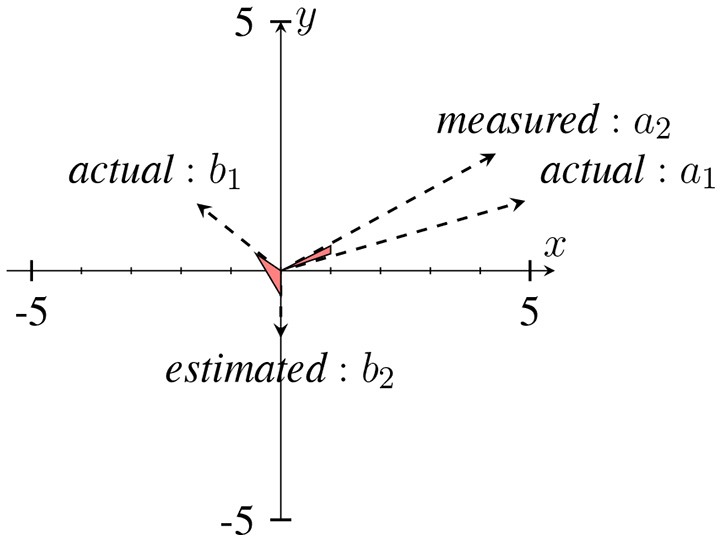
An example of a sudden phase change between *b*_1_ and *b*_2_ due to the low envelope in comparison to *a*_1_ and *a*_2_. The distance between (*a*_1_, *a*_2_) and (*b*_1_, *b*_2_) are approximately the same, however with different envelopes. This has been adapted from Mortezapouraghdam and Strauss (2017). Figure with some modifications has been adapted from Mortezapouraghdam and Strauss (2017).

In Sameni and Seraj (2016), it has been shown that as the IE becomes small (near zero), the IP will contain sudden changes or jitters (see Figure 4) that can be falsely correlated to phase-resets induced from an event. We can describe the effect of spurious phase variation in a small example as in Figure 5 using four samples of an analytic signal. The distance between the samples |*a*_1_ − *a*_2_| and |*b*_1_ − *b*_2_| are the same. However, the envelope, which can be represented as the distance of the individual samples to the origin are different. Considering *a*_2_ and *b*_2_ represent the noisy samples, the noise effect between the actual sample of *a*_1_ and *a*_2_ yields an angle of ~ 13°, where as in case of sample *b*, this is about 116°. Such variation in this example relates to the low envelope of the signal.

This effect has been studied in Chavez et al. (2006), Rudrauf et al. (2006), and Sameni and Seraj (2017) where the effect of spurious phase jumps have been described in terms of calculation of the phase. The IP is computed using the arctan operator $\left(\tan^{-1}\left(\frac{u\left(t\right)}{x\left(t\right)}\right)\right)$. Minor changes due to noise or background EEG variations to the real and imaginary parts of the analytic signal (which are narrow-bandpassed) can lead to significant changes to the computation of phase as the numerator and denominator values are small (see Rudrauf et al., 2006 and the references therein). Hence, the aim of this study is to estimate the *true phase* using the IE and IP and have a more reliable measure of the IP such that the spurious phase variations due to a low envelope will be removed.

### 2.2. Modeling the variations between instantaneous phase and amplitude

The analytic form of the signal *s*_*t*_ ∈ ℝ is denoted as ${\hat{s}}_{t}=s_{t}+iH\left(s_{t}\right),{\hat{s}}_{t}\in \mathbb{C}$. We assume that the measurements are corrupted by Gaussian noise with mean 0 and variance α. The noise of the measurements is denoted by *W*_*t*_. It's analytical form ${\hat{W}}_{t}$ is modeled using a symmetric complex Gaussian distribution ${\hat{W}}_{t}\sim \mathrm{CN}\left(0,\alpha I\right)$. This can be shown as follows: as stated, the bandpassed signal is modeled as

$$x_{t}=X_{t}\cos\left(\omega_{0}t+\theta_{t}\right)+W_{t}=s_{t}+W_{t}.$$

It's HT is denoted by

$$\begin{array}{l} H\left(x_{t}\right)=H\left(X_{t}\cos\left(\omega_{0}t+\theta_{t}\right)+W_{t}\right)\\ \;=X_{t}\sin\left(\omega_{0}t+\theta_{t}\right)+H\left(W_{t}\right) \end{array}$$

using the linearity of the HT. Here $H\left(W_{t}\right)$ is also a Gaussian distributed random variable (see chapter 8 of Davenport and Root, 1958; Sameni and Seraj, 2016) with mean 0 and variance α. The analytic form of the signal *x*_*t*_ using the HT is then

(1) $$\begin{array}{l} {\hat{x}}_{t}=x_{t}+iH\left(x_{t}\right)\\ \;=X_{t}\cos\left(\omega_{0}t+\theta_{t}\right)+W_{t}+i\left[X_{t}\sin\left(\omega_{0}t+\theta_{t}\right)+H\left(W_{t}\right)\right]\\ \;=X_{t}\left[\cos\left(\omega_{0}t+\theta_{t}\right)+i\sin\left(\omega_{0}t+\theta_{t}\right)\right]+\left[W_{t}+iH\left(W_{t}\right)\right]\\ \;=X_{t}{\text{e}}^{i\left(\omega_{0}t+\theta_{t}\right)}+\left[W_{t}+iH\left(W_{t}\right)\right] \end{array}$$

where the analytic form of *W*_*t*_ is modeled by a complex Gaussian distribution $W_{t}+iH\left(W_{t}\right)\sim \mathrm{CN}\left(0,I\alpha \right)\sim \mathrm{CN}\left(0,\left[\begin{array}{cc} \alpha & 0\\ 0 & \alpha \end{array}\right]\right)$ with a zero mean and independent real and imaginary part with σ^2^ as the variance^2^. The resulting complex Gaussian distribution is a symmetric distribution with non-diagonal elements set to zero.

The analytic form of the measurements are hence modeled as ${\hat{x}}_{t}={\hat{s}}_{t}+{\hat{W}}_{t}$, where ŝ_*t*_ ∈ ℂ is the analytic form of the signal without noise. We work directly with the complex analytic signals, as we can later obtain the denoised version of the IP to simplify our model. With our assumption of *s*_*t*_, the observations can be modeled using a complex Gaussian distribution ${\hat{x}}_{t}\sim \mathrm{CN}\left({\hat{s}}_{t},\alpha \right)$, where ŝ_*t*_ is the mean and α is the variance. The signal *s*_*t*_ is filtered with a narrow bandpass filter centered around *f*_*c*_ prior to computing the analytic signal ${\hat{x}}_{t}$ (see Equation 1). Thus we can assume that ${\hat{x}}_{t}=A_{t}e^{i\left(\omega_{0}t+\theta_{t}\right)}+{\hat{W}}_{t}$, using the linearity of HT. Here, ${\hat{s}}_{t}=A_{t}e^{i\left(\omega_{0}t+\theta_{t}\right)}$ is the denoised analytical signal that we want to recover. The ratio of successive values of ŝ_*t*_ is

(2) $$\begin{array}{l} {\hat{s}}_{t+1}/{\hat{s}}_{t}=\left(A_{t+1}/A_{t}\right)e^{i\left(\omega_{0}\left(t+1\right)+\theta_{t+1}-\omega_{0}t-\theta_{t}\right)}\\ \;=\left(A_{t+1}/A_{t}\right)e^{i\left(\omega_{0}+\left(\theta_{t+1}-\theta_{t}\right)\right)} \end{array}$$

(3) $$\begin{array}{l} \approx e^{i\omega_{0}} \end{array}$$

The results are obtained based on the assumption that at a narrow frequency, *A*_*t*_ and θ_*t*_ are varying slowly compared to ω_0_. We can therefore express the phase modulations over time using our assumptions as ${\hat{s}}_{t+1}\approx e^{i\omega_{0}}{\hat{s}}_{t}$, and model ${\hat{s}}_{t+1}=e^{i\omega_{0}}{\hat{s}}_{t}+{\hat{\eta }}_{t}$, where ${\hat{\eta }}_{t}\sim CN\left(0,\sigma \right)$. This means that the analytic signal at time *t* + 1 is obtained using the phase at time *t* multiplied by a small factor of $e^{i\omega_{0}}$ with some additive noise. The additive noise η correspond to the simplifications that have been applied for obtaining Equation (3). We assume that the additive noise follows a Gaussian distribution. The proposed model for the phase guarantees that the changes in phase of a narrow-bandpassed signal are rather slow and gradual over time.

#### 2.2.1. Model derivation

We describe the model derivation in two phases of forward and backward pass. The model assumptions as described previously are as follows:

(4) $$\begin{array}{l} {\hat{s}}_{t+1}=e^{i\omega_{0}}{\hat{s}}_{t}+\hat{\eta_{t}}\text{ with }\hat{\eta_{t}}\sim \mathrm{CN}\left(0,\sigma \right)\\ \;{\hat{x}}_{t}={\hat{s}}_{t}+{\hat{W}}_{t}\text{ with }{\hat{W}}_{t}\sim \mathrm{CN}\left(0,\alpha \right) \end{array}$$

with the following assumptions about the distributions of states and the data observations:

(5) $$\begin{array}{l} P\left({\hat{s}}_{1}\right)\;\sim \mathrm{CN}\left(\mu_{1},p_{1}\right)\\ P\left({\hat{s}}_{t+1}|{\hat{s}}_{t}\right)\;\sim \mathrm{CN}\left(e^{i\omega_{0}}{\hat{s}}_{t},\sigma \right)\\ P\left({\hat{x}}_{t}|{\hat{s}}_{t}\right)\;\sim \mathrm{CN}\left({\hat{s}}_{t},\alpha \right) \end{array}$$

We can write the above expressions as a *two dimensional real linear Gaussian* state-space model by separating the real and imaginary parts. In the following we take $\hat{s_{t}},\hat{x_{t}}$ to be two dimensional vectors of real numbers consisting of the real and imaginary parts of the underlying complex number. With this convention we have:

(6) $$\begin{array}{ll} P\left({\hat{s}}_{1}\right) & \sim N\left([\begin{array}{c} ℜ\left(\mu_{1}\right)\\ ℑ\left(\mu_{1}\right) \end{array}],[\begin{array}{cc} p_{1} & 0\\ 0 & p_{1} \end{array}]\right)\\ P\left({\hat{s}}_{t+1}|{\hat{s}}_{t}\right) & \sim N\left([\begin{array}{cc} \cos\left(\omega_{0}\right) & -\sin\left(\omega_{0}\right)\\ \sin\left(\omega_{0}\right) & \cos\left(\omega_{0}\right) \end{array}]{\hat{s}}_{t},[\begin{array}{cc} \sigma & 0\\ 0 & \sigma \end{array}]\right)\\ P\left({\hat{x}}_{t}|{\hat{s}}_{t}\right) & \sim N\left({\hat{s}}_{t},[\begin{array}{cc} \alpha & 0\\ 0 & \alpha \end{array}]\right) \end{array}$$

In this form we can directly apply the Kalman smoother that has been described in Briers et al. (2010). This consists of a forward and a backward pass which we describe in the following two sections.

##### The forward pass

To estimate the state (an estimation of the analytic form of the signal), we have to derive the following posterior distribution:

$$\begin{array}{l} P\left({\hat{s}}_{t}|{\hat{x}}_{1:t}\right)=\frac{P\left({\hat{x}}_{t}|{\hat{s}}_{t},{\hat{x}}_{1:t-1}\right)P\left({\hat{s}}_{t},{\hat{x}}_{1:t-1}\right)}{P\left({\hat{x}}_{1:t}\right)}\\ \;=P\left({\hat{x}}_{t}|{\hat{s}}_{t}\right)\frac{P\left({\hat{s}}_{t}|{\hat{x}}_{1:t-1}\right)}{P\left({\hat{x}}_{t}|{\hat{x}}_{1:t-1}\right)} \end{array}$$

by using the first order Markov property that the current data at time *t* is independent from the past given the state at time ŝ_*t*_. Simplifying the normalization factor we can write:

$$\begin{array}{l} P\left({\hat{s}}_{t}|{\hat{x}}_{1:t}\right)\propto P\left({\hat{x}}_{t}|{\hat{s}}_{t}\right)P\left({\hat{s}}_{t}|{\hat{x}}_{1:t-1}\right)\\ \;\propto P\left({\hat{x}}_{t}|{\hat{s}}_{t}\right)\underset{\text{Marginalizing over }{\hat{s}}_{t-1}}{\underset{︸}{\int P\left({\hat{s}}_{t},{\hat{s}}_{t-1}|{\hat{x}}_{1:t-1}\right)d{\hat{s}}_{t-1}}} \end{array}$$

after expanding the inner bracket, we obtain:

(7) $$\begin{array}{l} P\left({\hat{s}}_{t}|{\hat{x}}_{1:t}\right)\;\propto P\left({\hat{x}}_{t}|{\hat{s}}_{t}\right)\int P\left({\hat{s}}_{t}|{\hat{s}}_{t-1}\right)P\left({\hat{s}}_{t-1}|{\hat{x}}_{1:t-1}\right)d{\hat{s}}_{t-1} \end{array}$$

**Figure 6:**
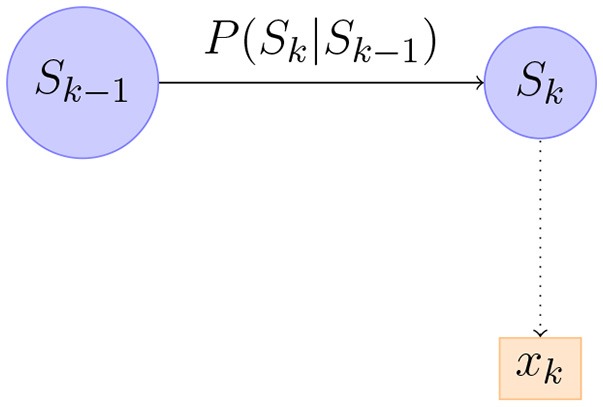
An illustration of part of a Bayesian Network corresponding to forward-passing of information.

Equation 7 can be realized as in Figure 6. Figure 6 shows a part of the first order Markov model. We can compute the distribution of $P\left({\hat{s}}_{t}|{\hat{x}}_{1:t}\right)$ recursively starting from *P*(ŝ_0_). It turns out that in our model the distribution of $P\left({\hat{s}}_{t}|{\hat{x}}_{1:t}\right)$ is always Gaussian so it suffices to compute its mean and covariance matrix. Writing μ_*t*_, *P*_*t*_ for the mean and covariance matrix of $P\left({\hat{s}}_{t}|{\hat{x}}_{1:t}\right)$ we have the following equations from Briers et al. (2010).

$$\begin{array}{l} \;P_{t}^{\prime }=BP_{t}B^{\text{T}}+Q\\ \;K=P_{t}^{\prime }{\left(P_{t}^{\prime }+R\right)}^{-1}\\ \mu_{t+1}=B\mu_{t}+K_{t}\left({\hat{x}}_{t}-B\mu_{t}\right)\\ \;P_{t+1}=P_{t}^{\prime }-K_{t}P_{t}^{\prime \text{T}} \end{array}$$

Furthermore, it turns out that *P*_*t*_ is always diagonal of the form *p*_*t*_*I*. This allows us to simplify the equations above. To see this, assume that *P*_*t*_ = *p*_*t*_*I* and plug this expression into the equations above.

$$\begin{array}{l} \;P_{t}^{\prime }\;=B\left(p_{t}I\right)B^{T}+Q\\ \;=p_{t}BB^{T}+\sigma I=\left(p_{t}+\sigma \right)I\\ \;K_{t}\;=\left(p_{t}+\sigma \right)I{\left(\left(p_{t}+\sigma \right)I+\alpha I\right)}^{-1}\\ \;=\frac{p_{t}+\sigma }{p_{t}+\sigma +\alpha }I\\ \;\mu_{t}\;=B\mu_{t}+\frac{p_{t}+\sigma }{p_{t}+\sigma +\alpha }\left({\hat{X}}_{t}-B\mu_{t}\right)\\ P_{t+1}=\left(p_{t}+\sigma -\frac{{\left(p_{t}+\sigma \right)}^{2}}{p_{t}+\sigma +\alpha }\right)I \end{array}$$

The *kalman gain factor* $\left(K_{t}=\frac{p_{t}+\sigma }{p_{t}+\sigma +\alpha }I\right)$ is an expression for determining how reliable the measurement at time *t* + 1 is compared to the estimated state (signal) based on the level of noise in the data. If data has a small level of SNR (high noise), the algorithm will rely more on the estimated value than the measurement. Therefore, a realistic and good estimate of SNR can improve the performance of the KS.

##### The backward pass

Given $P\left({\hat{s}}_{t}|{\hat{x}}_{1:t}\right)$ we compute $P\left({\hat{s}}_{t}|{\hat{x}}_{1:T}\right)$ recursively starting from $P\left({\hat{s}}_{T}|{\hat{x}}_{1:T}\right)$ and moving backwards. In general we have

(8) $$\begin{array}{l} P\left({\hat{s}}_{t}|{\hat{x}}_{1:T}\right)=P\left({\hat{s}}_{t}|{\hat{x}}_{1:t}\right)\int \frac{P\left({\hat{s}}_{t+1}|{\hat{x}}_{1:T}\right)P\left({\hat{s}}_{t+1}|{\hat{s}}_{t}\right)}{P\left({\hat{s}}_{t+1}|{\hat{x}}_{1:t}\right)}d{\hat{s}}_{t+1} \end{array}$$

and hence we can compute $P\left({\hat{s}}_{t}|{\hat{x}}_{1:T}\right)$ given $P\left({\hat{s}}_{t+1}|{\hat{x}}_{1:T}\right)$. Again, we find that $P\left({\hat{s}}_{t}|{\hat{x}}_{1:T}\right)$ is always a Gaussian distributed random variable. Writing ${\bar{\mu }}_{t},{\bar{P}}_{t}$ for the mean and covariance matrix of $P\left({\hat{s}}_{t}|{\hat{x}}_{1:T}\right)$ we have the following equations from Briers et al. (2010).

$$\begin{array}{l} \Gamma_{t}=P_{t}B^{\text{T}}{\left(p_{t}^{\prime }\right)}^{-1}\\ {\bar{\mu }}_{t}=\mu_{t}+\Gamma_{t}\left({\bar{\mu }}_{t+1}-B\mu_{t}\right)\\ {\bar{P}}_{t}\;=P_{t}+\Gamma_{t}\left({\bar{P}}_{t+1}-P_{t}^{\prime }\right)\Gamma_{t}^{\text{T}} \end{array}$$

As in the forward pass this simplifies in our model, since we only deal with symmetric covariance matrices of the form $\bar{p_{t}}I$ and since *B*^*T*^ = *B*^−1^. In the following we assume that ${\bar{P}}_{t+1}=\bar{p_{t+1}}I$. We have

$$\begin{array}{l} \Gamma_{t}=p_{t}IB^{\text{T}}{\left(\left(p_{t}+\sigma \right)I\right)}^{-1}=\frac{p_{t}}{p_{t}+\sigma }B^{\text{T}}\\ \bar{\mu_{t}}\;=\mu_{t}+\frac{p_{t}}{p_{t}+\sigma }B^{T}\left({\bar{\mu }}_{t+1}-B\mu_{t}\right)\\ \;=\mu_{t}+\frac{p_{t}}{p_{t}+\sigma }\left(B^{T}{\bar{\mu }}_{t+1}-\mu_{t}\right)\\ {\bar{P}}_{t}\;=p_{t}I+\Gamma_{t}\left({\bar{p}}_{t+1}-p_{t}-\sigma \right)\Gamma_{t}^{\text{T}}\\ \;=\left(p_{t}+\frac{p_{t}^{2}\left({\bar{p}}_{t+1}-p_{t}-\sigma \right)}{{\left(p_{t}+\sigma \right)}^{2}}\right)I \end{array}$$

The steps for applying a KS are shown in the algorithm below:

**Algorithm 1:**
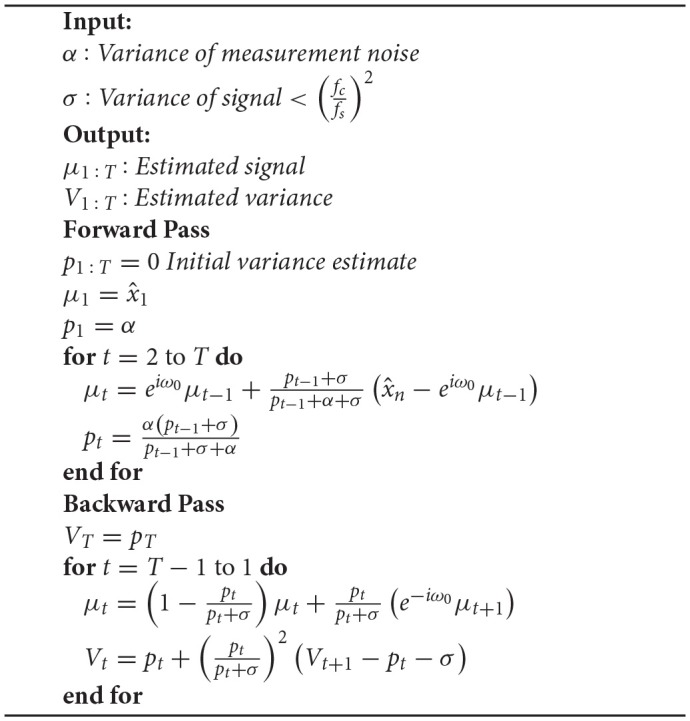
Algorithmic representation of a Kalman smoother (The forward and backward pass)

## 3. Results

### 3.1. Analysis of synthetic generated spurious phase variation

**Figure 7:**
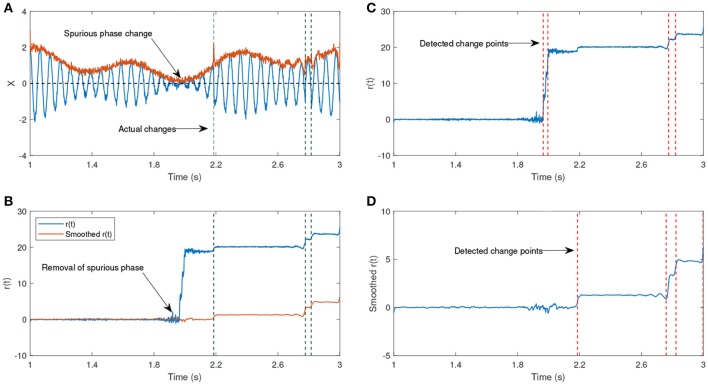
**(A)** An example of an oscillatory signal which contains a low envelope. The signal length is about 2 s. The vertical lines show the actual shifts in the signal which are random. **(B)** The phase after removing the line corresponding to the center frequency before and after applying a Kalman smoother (KS). **(C)** The detected changes in r(t) without any smoothing have been plotted. **(D)** The detected changes in r(t) after applying KS. **(A,B)** Have been adapted from Mortezapouraghdam and Strauss (2017) with some modifications.

In Figure 7, we show an example of an amplitude modulated signal with phase reset at the time step *t* = 2.18*s*. The signal has been generated according to

(9) $$\begin{array}{l} X\left(t\right)=\{\begin{array}{cc} \left[\cos{\left(\omega_{0}t\right)}^{2}+\epsilon \right]\cos\left(\omega_{1}t\right)\; & \text{if }t<u\\ \left[\cos{\left(\omega_{0}t\right)}^{2}+\epsilon \right]\cos\left(\omega_{1}t+\pi \right) & \text{otherwise} \end{array}. \end{array}$$

The signal generated after the time step *u* is shifted by π, hence the time step *u* is the actual change point. The parameters ω_0_ and ω_1_ have different frequencies, such that one of them corresponds to the generation of a lower amplitude signal. The shift after the time step *u* will lead to a phase reset that is related to the signal and we are interested to track this change over time. The other phase jitters which we aim to remove correspond to the low envelope of the signal. Figure 7b shows the estimated phase before and after applying the KS. Before applying KS, there are two phase jitters due to the low envelope and the actual shift in the signal. After applying the KS, the jitter corresponding to the low envelope has been diminished. In addition, the standard deviation of the estimated phase is an indicator for the degree of reliability of the phase jitter. A low standard deviation in the estimated signal phase indicates that it is less likely that the phase variation is due to the low envelope, whereas a high standard deviation indicates a higher likelihood that the phase variation has been generated due to noise.

In this example, the standard deviation from the sample *t* = 300 increases, indicating a high uncertainty due to the artificial phase reset. To assess the accuracy of the model, we generated 500 synthetic time-series with different levels of added noise and random shifts in the signal. For every number of change points *n*, we randomly shift the signal at *n* random time steps *u* between $\frac{\pi }{8}$ and $\frac{7\pi }{8}$. This generates *n* instances at which the signal has been shifted, and the other phase variations correspond to either the noise or low envelope of the signal (see Figure 7).

We apply a KS to remove the noisy variations in the signal. To get an estimate of how reliably the change points have been removed, we apply a change point algorithm to detect the time steps that the mean of the signal has been significantly altered^3^. The detected time instances are recorded as the estimated significant change points before and after applying KS (see Figure 7). If the *difference of the estimated change points* and *the actual random change points* are less than 10 samples, the estimated change point is assumed to be correct (*true positive, TP*). However, if the difference is larger than the determined threshold, the data point has been *falsely recognized* as a change point (*and is referred to false positive, fp*). If the change point algorithm fails to detect the actual change point, then the point is referred to as *false negative, fn*.

**Figure 8:**
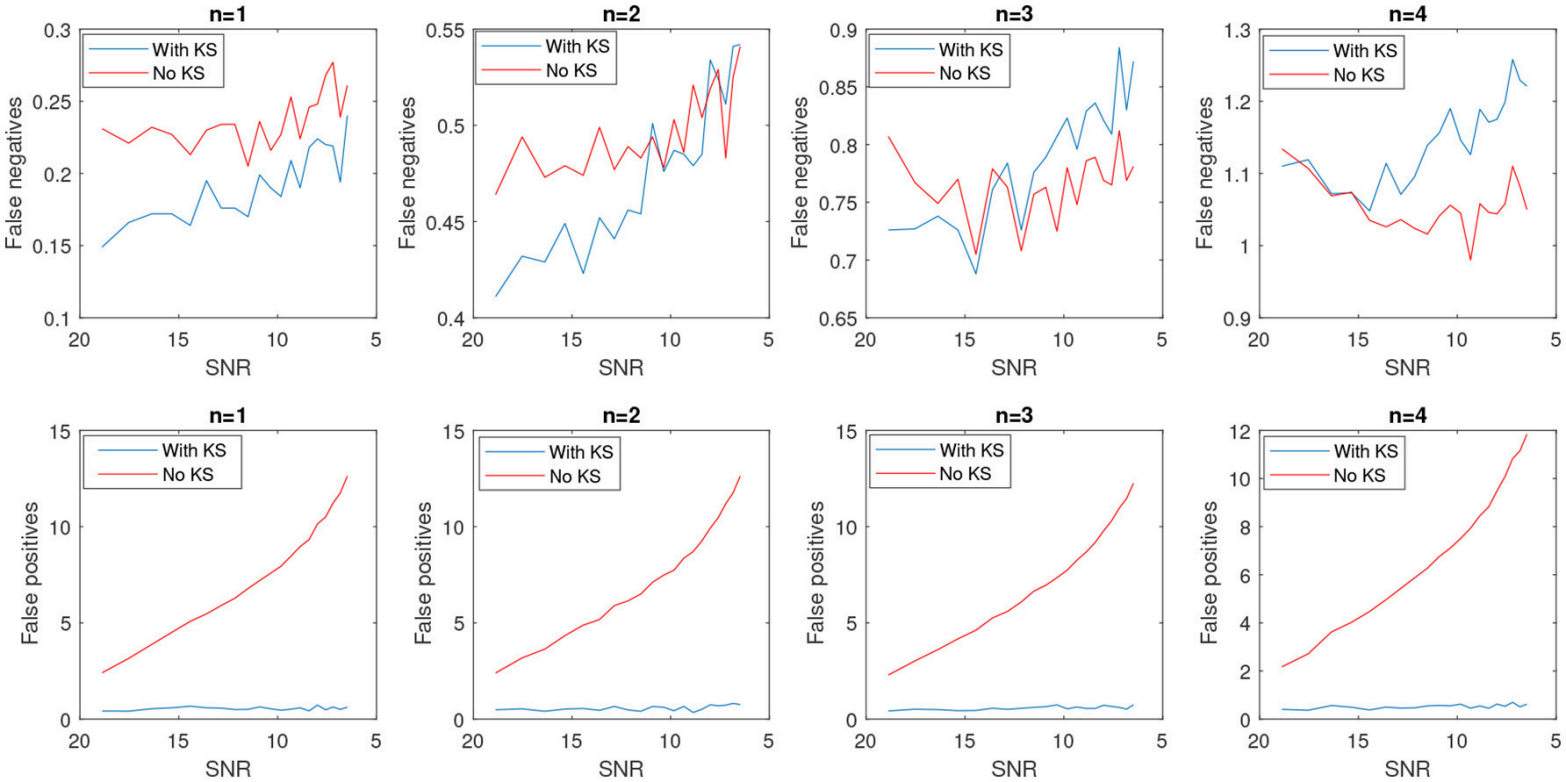
Average number of false positives and false negatives for 2,000 batch of synthetic time-series signals with length of 1,500 samples. The number of actual changes in the signal are indicated as *n* and results are shown for an increasing SNR.

In Figure 8, the average number of false positives (*fp*) and false negatives (*fn*) for different number of change points have been plotted. For every number of change points *n*, we generated 2000 batches of data with different levels of noise and reported the average number of *fn* and *fp*. *After applying KS, the average number of fps is significantly reduced*. However, the case with no filtering yields very unstable results as the noise level increases. The average false negatives is however lower for the case that we apply no smoothing compared to results after KS. This is mainly due to the fact that more random changes are detected in the pre-smoothing condition. Therefore, as many indices will be assigned correctly as change points, satisfying the minimum distance criteria. In the case of smoothing, the overlap of a change point and low envelope can cause an increase in the number of detected false negatives. Using the measured rates of false negatives and positives, we computed the *Matthews correlation coefficient, MCC*. MCC takes the number of false/true positive/negatives and returns a correlation coefficient between the observed and predicted binary classifier. It is computed as

$$\begin{array}{l} MCC=\frac{TP.TN-FP.FN}{\sqrt{\left(TP+FP\right)\left(TP+FN\right)\left(TN+FP\right)\left(TN+FN\right)}}. \end{array}$$

**Figure 9:**
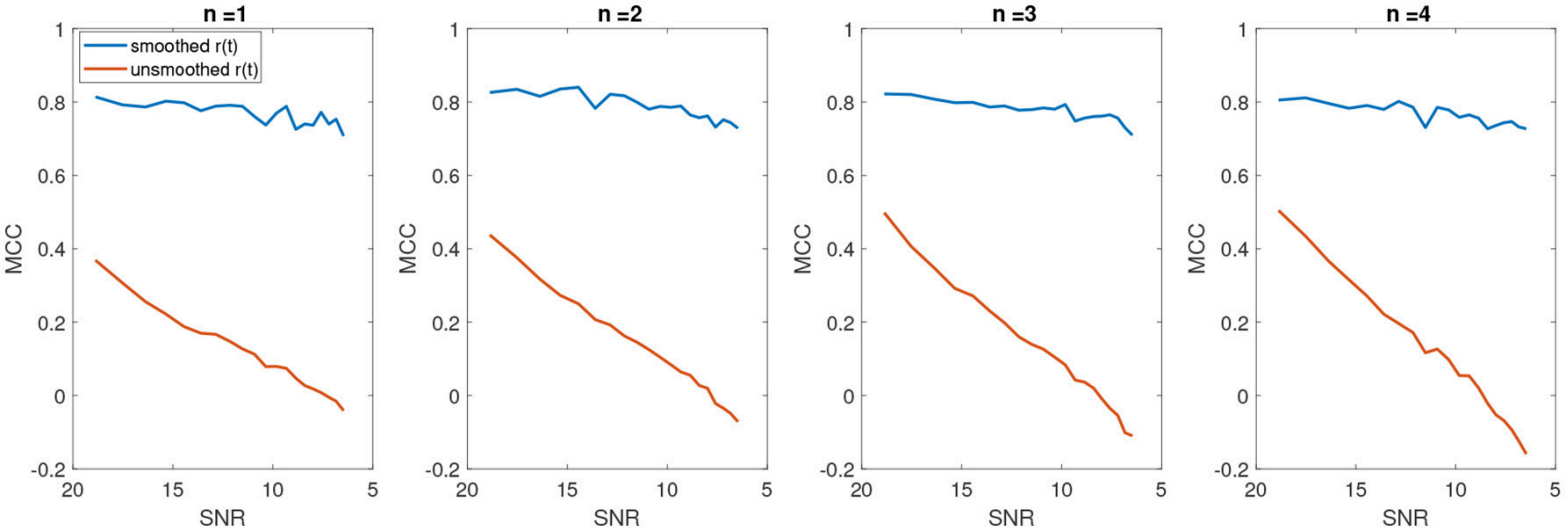
The average Matthews correlation coefficient for different number of SNRs and different number of change points.

A coefficient of +1 indicates a perfect prediction (i.e., in our case a perfect detection of change points at the correct indices), zero indicates no better than random assignment of change points and a coefficient of -1 means a complete disagreement between the predicted change points and the actual ones. Figure 9 shows the average MCC for different SNRs for varying change points *n*. The MCC significantly decreases as the SNR increases (as noise level decreases), indicating a random assignment of change points. This is also consistent with the average results of falsely detected change points. As the number of falsely detected change points increases, we have a more random assignment of change points. This is however not the case for the post-KS condition. The higher average of MCC indicates a significant improvement in the accuracy of detected change points.

### 3.2. Analysis of IP of synthetic EEG signal

In this section we generate additional synthetic signals for testing the effect of spurious phase variations. The synthetic signals are generated as a superposition of sinusoidal signals and noise with different amplitudes^4^. The signals has been narrow-bandpassed using a FIR filter to a center frequency of 7.6 Hz. The goal is to analyze the effect of the KS on removing the spurious phase variation for regions where the envelope is low. In this case, we considered the IP of signal corresponding to envelopes below 0.2 to be noise, and therefore need to be removed.

**Figure 10:**
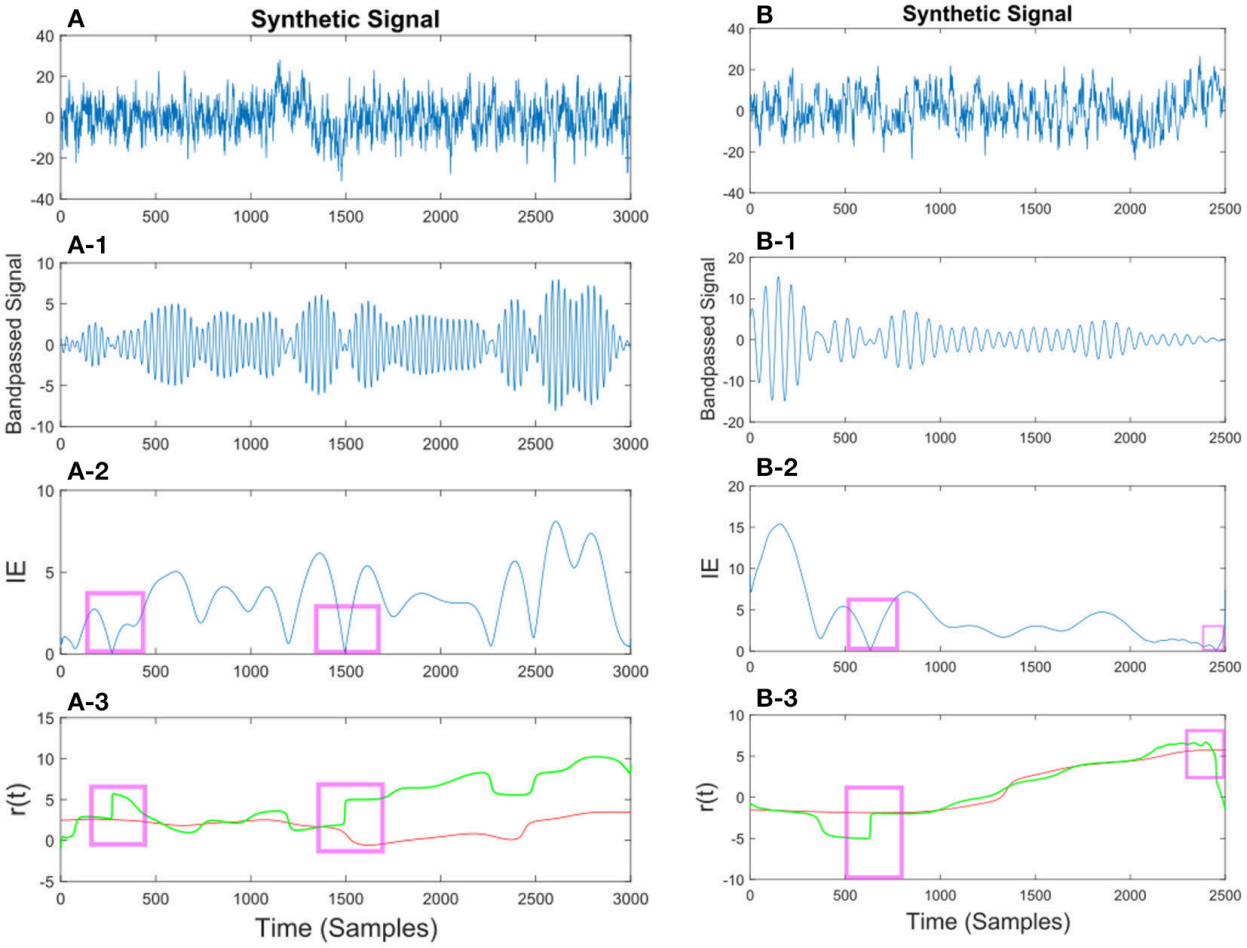
**(A)** Synthetic EEG signal generated at a *f*_*s*_ = 250Hz with a SNR of 0.11 (high noise variance) bandpassed at *f*_*c*_ = 7.6 Hz. Different noise amplitudes have been used for the two examples. The instantaneous envelope (IE) has been plotted with red boxes showing the regions with a low envelope below 0.2. In the last plot the resulting *r*(*t*) has been plotted for the smoothed (red) and non-smoothed (green) signal. The red regions show the effect of the smoothing on the regions with a low IE. **(B)** Same description as in **(A)** applies to **(B)** with a SNR of 0.13.

**Figure 11:**
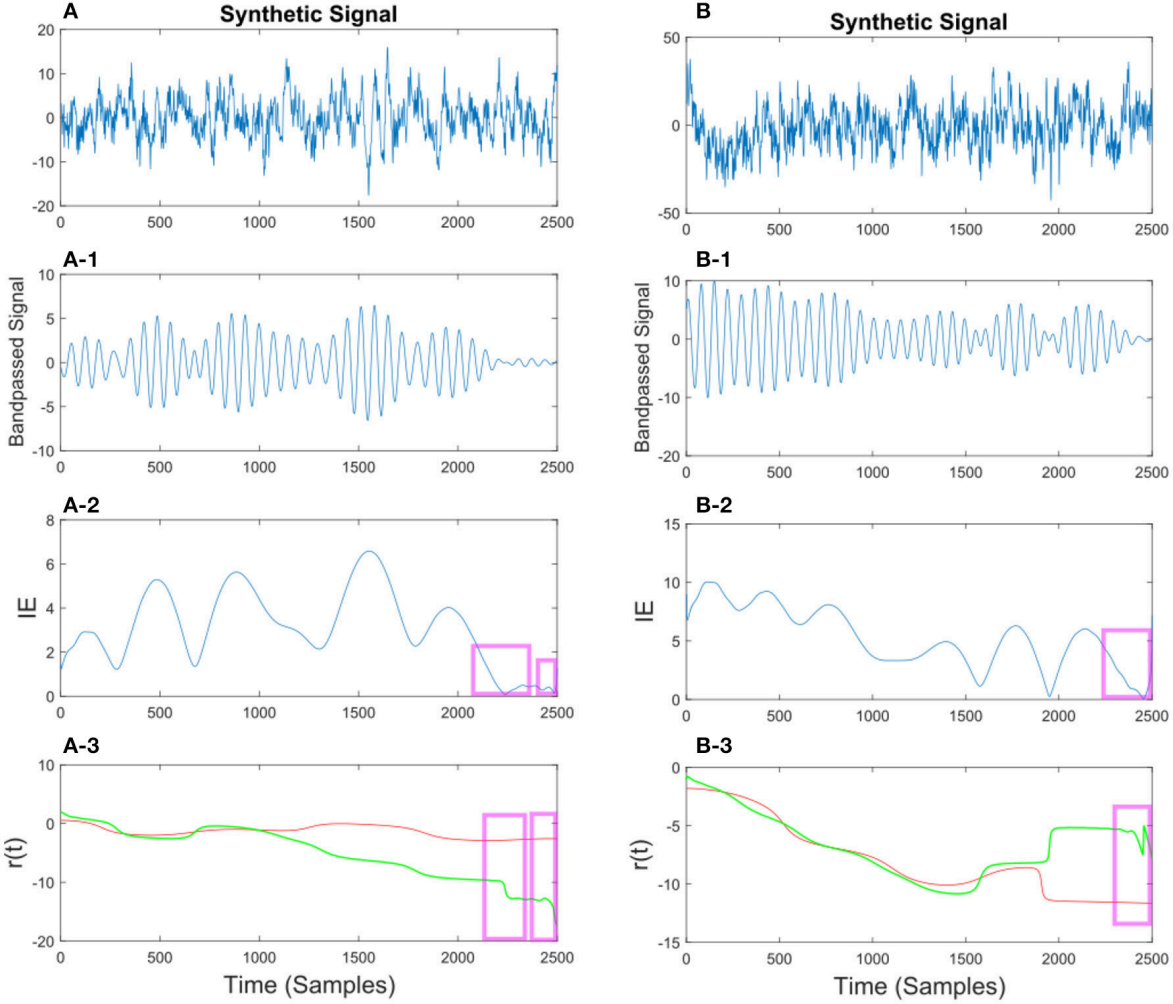
**(A)** Synthetic EEG signal generated at a *f*_*s*_ = 250 Hz with a SNR of 0.43 bandpassed at *f*_*c*_ = 7.6 Hz. The instantaneous envelope (IE) **(A-2)** has been plotted with red boxes showing the regions with a low envelope below 0.2. In the last **(A-3)** plot the resulting *r*(*t*) has been plotted for the smoothed (red) and non-smoothed (green) signal. The red regions show the effect of the smoothing on the regions with a low IE. **(B)** Same description as in **(A)** applies to **(B)** with a SNR of 0.047.

In Figures 10, 11 we show different examples of synthetic EEG signals where at some instances the corresponding envelope of the band-passed signal approaches zero. The regions corresponding to the low envelope has been illustrated in a red box. We show how applying the method with proper set of parameters can remove the spurious changes in the IP of the signal. The main incentive is to remove the spurious variations in *r*(*t*). As we are considering the phase information in a narrow-bandpass, we are required to filter the data accordingly. In section 3.4 we describe in more detail on the choices of parameter setting.

### 3.3. Application of KS on EEG recordings

**Figure 12:**
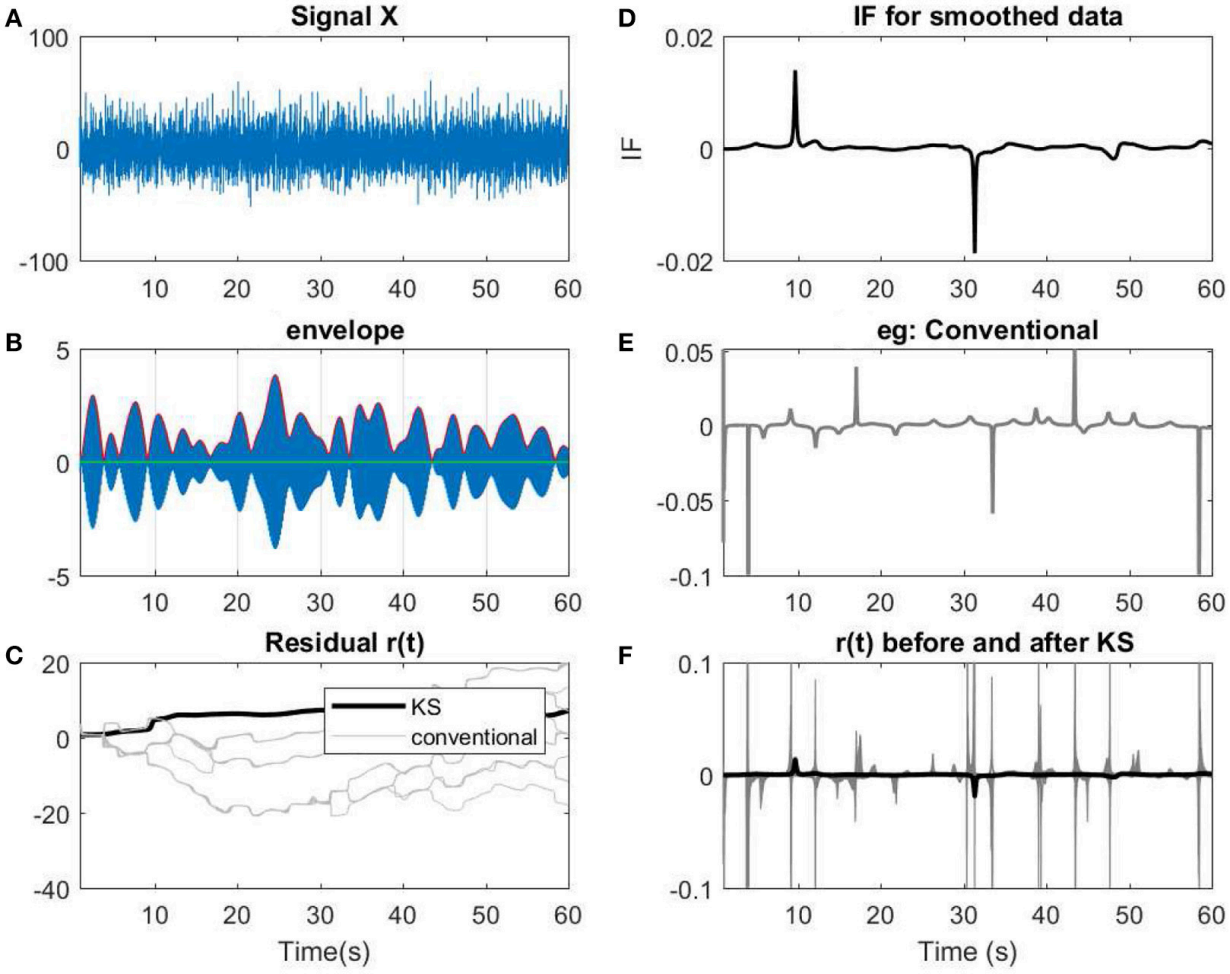
**(A)** A 60 s segment of measured EEG data. **(B)** Data has been narrow band-passed with a center frequency of 7 Hz. The envelope of the analytic band-passed signal has been plotted in red. **(C)** The residual of IP before and after applying KS has been applied. The term conventional corresponds to the filtered signals using a narrow band-pass filter before applying KS. As described previously, for every filter, a slight perturbation has been applied to the filter. **(D)** The IF of the smoothed data. **(E)** The resulting IF of one of the instances of the filtering process that has been applied on the data. As shown, in the conventional method, more spikes or variations are observed in te IF. **(F)** Comparing the IF of smoothed narrow-bandpassed signal (dark line) with IF of all the non-smoothed signals. The changes in regard to low envelopes in **(B)** can be observed as fewer abrupt changes after applying KS in the smoothed version than the conventional approach.

**Figure 13:**
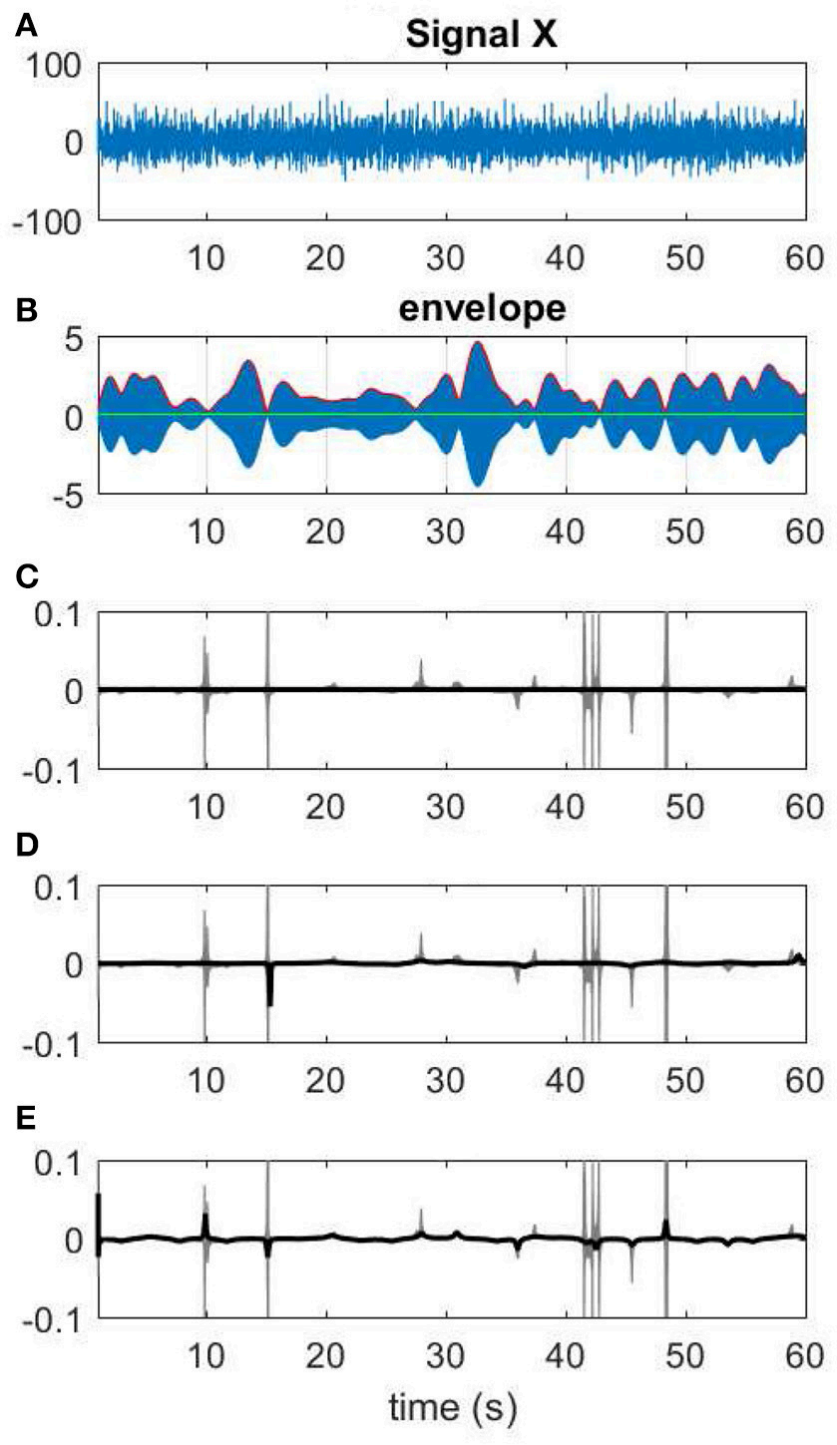
Different choices of Kalman smoothing factor. **(A)** A 60-s segment of EEG measurement. **(B)** The narrow band-passed signal along with its envelope. **(C)** The extreme case of zero factor yields a line for IF. **(D)** A factor of 0.005. **(E)** IF resulting from a high factor >100.

In this section, we apply the proposed model on few examples of an EEG recording (See Figures 12, 13). The recording measurements are obtained from the right and left mastoid electrodes that have been obtained during an experimental listening paradigm (for more information about the data measurement and the details see Corona-Strauss and Strauss, 2017). The EEG signals were bandpassed between 1–70 Hz. In order to test the applicability of the model, we need to narrow-bandpass the signal and then estimate the IP of the filtered data.

We compute the IP using the analytic form of the signal with the Hilbert transformation. To have a meaningful interpretation of the IP, the signal is narrow-bandpassed around a certain center frequency of interest. A zero-phase forward backward IIR elliptic filter is used to narrow-bandpass the signal. As noted in Seraj and Sameni (2017), in many studies a FIR filter is used to avoid phase distortion, however as the signal is narrow-bandpassed, the order of the filter can significantly increase which leads to long transient response episode. The transient response are usually discarded for analysis. We therefore applied an IIR filter which requires a much lower order than a FIR filter and a zero-phase forward backward filtering ensures a zero-phase distortion.

In Sameni and Seraj (2016), it is shown that variations to the filter parameters in filtering process can lead to changes in the IP and IF responses. Therefore, a robust estimation method that estimates the IP from the average ensemble of infinitesimal perturbations to the filter parameters is presented by Seraj and Sameni (2017). To estimate the IP, we apply a narrow-bandpass filter with slight variations in the frequency range for *M* = 100 iterations. At every iteration, the filter parameters are as follows: the filter order is 6, the reduction in the stop-band is 50dB, the ripple-passband is 0.01 and the filter bandwidth is set to 0.5. The center frequency is *f*_*c*_ = 7.4Hz and at every iteration, a slight perturbation is being applied to the center frequency. It has to be noted that the changes to filter parameters are very small that the effect is irrelevant for the study of most physiological effects.

### 3.4. The setting of the parameters

One of the important settings in using the proposed algorithm is the setting of the variance of noise of the measurement and signal (α and σ, respectively). In the examples provided in Figures 7 10, 11, we have access to the original signal without noise. This is equivalent to having prior knowledge of the distribution or shape of the signal for real scenarios. However, in most cases, we don't have access to the actual distribution of the signal and hence a proper *estimation* of the parameters is required. In this section we give suggestions for setting the parameters of the KS.

As described in Equation (4), the additive noise of the signal and measurement are defined as ${\hat{\eta }}_{t}\sim N\left(0,\sigma \right)$ and ${\hat{W}}_{t}\sim N\left(0,\alpha \right)$. By reordering ${\hat{s}}_{t+1}+e^{i\omega_{0}}{\hat{s}}_{t}+{\hat{\eta }}_{t}$, we have ${\hat{\eta }}_{t}={\hat{s}}_{t+1}-e^{i\omega_{0}}{\hat{s}}_{t}$. Therefore the variance of noise of the signal σ can be estimated as the variance of the difference equation of the analytic signal. In case of real measurements, as we don't have access to the actual signal, we can estimate the variance of the noise as follows:

(10) $$\begin{array}{l} {\hat{x}}_{t+1}-e^{i\omega_{0}}{\hat{x}}_{t}={\hat{s}}_{t+1}+{\hat{W}}_{t+1}-e^{i\omega_{0}}\left({\hat{s}}_{t}+{\hat{W}}_{t}\right)\\ \;={\hat{s}}_{t+1}+{\hat{W}}_{t+1}-e^{i\omega_{0}}{\hat{s}}_{t}-e^{i\omega_{0}}{\hat{W}}_{t}\\ \;=\underset{\eta_{t}}{\underset{︸}{{\hat{s}}_{t+1}-e^{i\omega_{0}}{\hat{s}}_{t}}}+\left({\hat{W}}_{t+1}-e^{i\omega_{0}}{\hat{W}}_{t}\right) \end{array}$$

As stated earlier *W*_*t* + 1_ and *W*_*t*_ are samples from a Gaussian distribution with a variance of α:(N(0,αI)). Due to symmetry of the Gaussian distributions, the sum of two normal distribution is a Gaussian with the summation of the means and the variances. Therefore, in our case, the resulting distribution will be N(0,2αI). By taking the variance of both sides in Equation (10), we have

$$\begin{array}{l} var\left({\hat{x}}_{t+1}-e^{i\omega_{0}}{\hat{x}}_{t}\right)=var\left(\eta_{t}\right)+var\left({\hat{W}}_{t+1}-e^{i\omega_{0}}{\hat{W}}_{t}\right)\\ \;=var\left(\eta_{t}\right)+2\alpha \end{array}$$

Hence the variance of noise of the signal can be estimated as $var\left(\eta_{t}\right)=var\left({\hat{x}}_{t+1}-e^{i\omega_{0}}{\hat{x}}_{t}\right)-2\alpha$. Setting $\epsilon =var\left({\hat{x}}_{t+1}-e^{i\omega_{0}}{\hat{x}}_{t}\right)$, the optimal σ is in the range σ ∈ [max(ϵ − 2α, 0), ϵ] where ϵ is the upper-bound of our estimation. The input for EEG measurements is ${\hat{X}}_{t}$ that is the average over all M filters. Another possibility for controlling the level of smoothing is to optimize over β*var*(η_*t*_) where β is a free parameter that controls the degree we rely on the measurements over the predictions. In the extreme case of β = 0, the KS will remove all variations in IP which can be observed as a straight line in the corresponding IF. This has been shown in Figure 13 where different KS parameters are used for smoothing the data.

The setting of α is based on the study that is presented in Sameni and Seraj (2016). We set the variance of the measurement noise to be the variance of the M estimated phases obtained from infinitesimal perturbations to the filter parameters. The signal that we use at the end is the ensemble average of the bandpassed signals. The setting of the parameters rely mostly on the application and condition of the experiment. If data is prone to a lot of noise sources, we need to smooth out the noise more, therefore higher β values are used. In case of a classification task between different neural cognitive processes, we can optimize the range of σ such that the distance between the analyzing measure of the two processes is maximized. In general, setting of the KS parameters require more investigation given the specifics of the experiment and its goals.

## 4. Discussion

In order to investigate phase of neural oscillatory activities in general, the need of better analytic machinery to exactly characterize the phase is high. The general limitations can come from the preprocessing of wide-band neural signals being recorded, and phase computation methods themselves. By taking a parametric approach, we focused on removing the spurious phase variations that can produce misleading or in-comprehensive results.

We apply a linear Gaussian state space model to the dynamics of the analytical signal at a narrow band around a specific frequency. The method was tested on synthetic signals as well as variants of EEG signals where low envelopes were considered as potential instances of spurious phase variations. Given the flexibility in the model parameters (α and σ) we are able to remove the jitters in phase as the envelope approaches zero.

In the current approach, the specific setting of the σ and α parameters are important for removing the spurious phase variations as they are used for computing the Kalman gain factor. The Kalman gain factor determines how strongly the model relies on its own prediction over the measurement. In this framework, we needed to assume that α was constant throughout the signal. However, in real EEG applications or neural oscillatory activities in general, the noise can be varying over time due to different sources of artifacts and the signals have a strong non-stationary behavior, especially around the event times of scientific interests. For example, β oscillation is known to increase its amplitude around the external visual or auditory cues and to decrease behavioral onset (e.g., movement onsets) in various cortical areas (Takahashi et al., 2015; Watanabe et al., 2015; Noda et al., 2017; Rule et al., 2017), while the phase dynamics locked to the sensory cues or behavioral onsets where the temporal evolution of the phase dynamics remain unclear (Rubino et al., 2006; Takahashi et al., 2011, 2015; Watanabe et al., 2015; Rule et al., 2017). A particular challenge to characterize phase oscillation dynamics is one of the motivations for our current work - how to precisely characterize oscillation phase when the amplitude of the oscillation is attenuated. Successful characterization of such transient dynamics will unveil underlying neural mechanism that is responsible for neural oscillation in general.

We therefore aim to additionally investigate the effect of a varying α in time and its impact on the smoothing procedure. We will also investigate the impact of the proposed method in different experimental settings where IP is heavily used for classification between different neural processes. In many past studies (Strauss et al., 2008; Mortezapouraghdam et al., 2015), the phase locking of IP has been used as an indicator for separating the presence and absence of attentional-binding due to different auditory stimulations. We therefore require proper methods to measure the level of phase locking of the neural activities in response to different stimulations and denoising the spurious phase variations by optimizing over KS parameters such that the phase resets bounded to neurological activities are preserved. In this regard, the overlap of the IP resets due to neural activities with the ones due to low envelope has to be investigated.

Furthermore, thanks to recent interest to investigate spatiotemporal dynamics of neural oscillaations using various types of array recording methods to simultaneously capture dynamics of evoked responses or phase variations across multiple channels over space, mostly horizontally, the demand to precisely characterize phase of neural oscillation has been arising (Liotti et al., 2010; Takahashi et al., 2011, 2015; Keane and Gong, 2015; Moon et al., 2015; Watanabe et al., 2015; Rule et al., 2017; Denker et al., 2018) using various types of multiple channel devices such as EEG, ECoG, or intracortical arrays. Although these recent work or methods developed therein can characterize various spatiotemporal patterns of oscillation activities, but those results are heavily relying on oscillation phases that are not as faithfully computed as in our current work.

Moreover, another trend in neuroscience is to characterize variability of neural signals in single trials or attempt to relate between neural variability and sensory/motor variations being observed (Matsuoka, 1990; Szymanski et al., 2011; Matsuo et al., 2013; Cui et al., 2016; Barczak et al., 2018; Dechery and MacLean, 2018). Therefore, we would like to extend our current method to characterize single trial neural oscillation data as well.

## 5. Conclusion

One of the main incentives of the current study is to remove the spurious variations in IP for a more reliable assessment of phase information. We present a model based on a Kalman smoother that models the variations of phase in a narrow-bandpassed signal. We evaluated the model for synthetic signals with spurious and actual phase jitters. We added different level of noises to signals and evaluated the number of true and false positives as an indicator for correct detection of actual phase jumps. Results show a significant improvement in reducing the number of false positives. The method is also applied on synthetic EEG signals generated as the superposition of sinusoidal waves with noise to assess the removal of spurious phase variations. Inspecting on various settings the method is able to remove the rapid transitions in phase that correspond to a low envelope. In both cases of known and unknown underlying phase shifts, an estimation to the variance of signal and noise measurements has been presented. We use the same approach on ongoing EEG recordings for testing the applicability of the approach. The proposed approach shows success in removing the spurious phase variations corresponding to a low envelope.

## Ethics statement

The design of the experiment was planned and in accordance with ethics guidelines and the Declaration of Helsinki and the study was also approved as a scientific study by the local ethics committee [Arztekammer des Saarlandes (Medical Council of the Saarland), Germany]. Every person had the free choice to abandon the procedure and withdraw their participation at any time.

## Author contributions

FC-S and DS were both involved in implementation of the study, measurements, and discussions. ZM is involved in proposing the methodology part of the study and analysis of data. KT has participated in the discussion of the results, future directions of the study, and contributing in the revision of the paper in its accepted form. All authors participated in the discussion of the results.

### Conflict of interest statement

The authors declare that the research was conducted in the absence of any commercial or financial relationships that could be construed as a potential conflict of interest.

## Footnotes

^1^The measurements are derived from a repetition of the seminal dichotic tone detection experiment of Hillyard et al. (1973) which demonstrated the N1–effect of late auditory evoked responses for the first time and their relation to early auditory selective attention. In a study conducted by Corona-Strauss and Strauss (2017), they report on the analysis of the collected data using different circular analysis techniques to the (Hardy space projected) segmented ERP and unsegmented ongoing EEG data suggesting a unified framework to analyze neural correlates of selective attention in ERPs and their ongoing EEG activity.

^2^For simplicity of notation, we write CN(a,b) instead of CN(a,Ib) in the rest of the paper.

^3^The function *findchangepts* in MATLAB using the *mean* returns the time instances at which the mean of the data changes abruptly.

^4^The code for generating the signals has been mainly used from https://github.com/pchrapka/phasereset with slight variation.
